# Concerns on the Effects of Electrode Positions in Electrolyte Container for the Oxygen Evolution Reaction

**DOI:** 10.3390/molecules28248143

**Published:** 2023-12-18

**Authors:** Fan Zhang, Yayun Zhao, Xiaofeng Chen, Shengxiao Zhao, Junjie Zhou, Zhiyi Lu, Yichao Lin

**Affiliations:** 1 Renewable Energy Engineering Institute, Power China Huadong Engineering Corporation Limited, Hangzhou 311122, China; zhang_f10@hdec.com (F.Z.); chen_xf2@hdec.com (X.C.); zhao_sx@hdec.com (S.Z.); 2 Key Laboratory of Advanced Fuel Cells and Electrolyzers Technology of Zhejiang Province, Ningbo Institute of Materials Technology and Engineering, Chinese Academy of Sciences, Ningbo 315201, China; zhoujunjie@nimte.ac.cn (J.Z.); luzhiyi@nimte.ac.cn (Z.L.); 3 Key Laboratory of Far-Shore Wind Power Technology of Zhejiang Province, Hangzhou 311122, China; 4 University of Chinese Academy of Sciences, Beijing 100049, China

**Keywords:** electrochemical, oxygen evolution reaction, three-electrode system, positions, concentration boundary layer

## Abstract

Water electrolysis is currently a major technique to produce clean hydrogen, which is regarded as a promising and sustainable energy carrier. The efficiency of water electrolysis is highly dependent on the oxygen evolution reaction (OER) on the anode. The evaluation of an OER electrocatalyst is frequently carried out on a three-electrode system in a container of electrolyte. Herein, we found that the electrode positions in the electrolyte container could significantly affect the data acquisition of OER performance. After a detailed investigation, we reveal that the difference of the OER activity of an electrocatalyst at a different position is originated from their different *iR*_u_ drop and the gas diffusion resistance. For the first time, this work evokes concerns on the accurate evaluation of electrocatalysts regarding the electrode position. For fair comparisons and reliable results, it is strongly suggested to keep the electrode position unchanged in the electrochemical measurements. In addition, the establishment of a standard electrolyzer setup for electrocatalysis evaluation in the electrochemical community is also called for.

## 1. Introduction

Hydrogen, as a sustainable energy carrier, possesses broad and abundant resources and high energy density and is not constrained by geographical limitations, thus distinguishing it from other renewable energy technologies such as tidal, solar, and wind energy [1,2]. Furthermore, the combustion of hydrogen only generates water as a byproduct, thereby not only addressing energy-related concerns but also mitigating environmental issues associated with conventional fossil fuels [3,4]. Within the hydrogen energy technology chain, hydrogen production plays a pivotal role and water electrolysis exhibits the potential for large-scale hydrogen generation while also enabling coupling with other forms of renewable energy for hydrogen–electric cycles [5]. Water electrolysis produces hydrogen on the cathode, while its efficiency is highly dependent on the oxygen evolution reaction (OER) on the anode. Currently, the sluggish kinetics of electrochemical OER hampers the widespread application of water electrolysis technology [6,7]. To date, substantial studies have been devoted to exploring efficient OER electrocatalysts, focusing on the subsequent three areas: (1) the design and optimization of catalysts with high OER efficiency, which play a crucial role in the OER reaction, including the exceptionally superior noble-metal-based metallic catalysts (such as IrO_2_ and RuO_2_) and some materials with unique electronic structures and chemical properties, such as cobalt-based and nickel-based catalysts, and so on [8,9,10,11,12,13,14,15,16,17]; (2) in-depth study of OER mechanisms and the detailed processes, including the formation and transformation of intermediates, as well as the reaction pathways and rate-determining steps, which have been revealed by utilizing advanced experimental techniques and theoretical calculations, thereby providing important theoretical foundations for guiding the design and optimization of catalysts [18,19,20,21,22,23]; (3) optimization of efficient and stable electrolyte systems, including electrolyte types (e.g., the electrolyte cations) and important parameters (e.g., pH value and composition of the electrolytes) [24,25,26,27].

However, OER in practical conditions exhibits remarkable complexity and remains inadequately understood, despite more than a century of study. Part of the reason is that the inconsistency of some easily overlooked experimental and sample-related factors during testing make the results poorly repeatable or make the interpretation of resulting responses challenging. For example, Boettcher and colleagues found that the reference electrode (RE) to working electrode (WE) distance is positively correlated with the value of the uncompensated resistance (*R*_u_) for a Au electrode (0.8 cm^−2^) with or without a Ni(Fe)O_x_H_y_ catalyst film [28]. Minor errors in *R*_u_ may lead to significant variations in data analysis, particularly at high current levels. They also pointed out that the WE and the counter electrode (CE) positions would have an impact on the magnitude of *R*_u_.

Herein, we found that the electrode position in the electrolyte also has a significant effect on the OER data. To our knowledge, there is no previous report concerning this issue. Specifically, we used a three-electrode system, including Ni sheet as the WE, Pt mesh as the CE, and Hg/HgO as the RE, to investigate the effects of three-electrode system positions in the container on the alkaline OER. The variations in the current densities of the electrodes at different positions in two circular electrolytic containers with different diameters were recorded by chronoamperometry, and the polarization curves were obtained by linear sweep voltammetry (LSV). We reveal that the difference in the OER activity at different positions is caused by their different solution resistances due to the concentration boundary layer of electrolyte and their different gas diffusion resistance.

## 2. Results and Discussion

**Figure 1:**
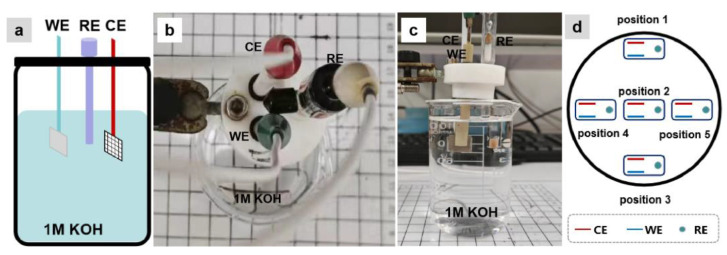
(**a**) Schematic of the container setup with WE, CE, and RE in 1 M KOH aqueous solution; (**b**) top-view photo and (**c**) side-view photo of the three-electrode system in the small electrolyte container; (**d**) the position trajectory of the three-electrode system in the small circular container (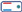: the three-electrode system).

During the electrochemical testing, we kept the three-electrode system as an integrated unit. This implies that the distances of WE to RE, WE to CE, and RE to CE were fixed, and the Ni sheet (WE) was kept parallel to the Pt mesh (CE), as shown in Figure 1a–c. We initially used a small cylindrical container with a diameter of ~5.5 cm and a height of ~7.5 cm as the electrolyte container. The three-electrode system was placed into the electrolyte container, with the WE and CE aligned along a central axis of the container. The electrode position with the CE near and approximately parallel to the container wall served as the starting point (position 1), and then the container was moved to change the position of the three-electrode system in the container according to the position trajectory shown in Figure 1d.

**Figure 2:**
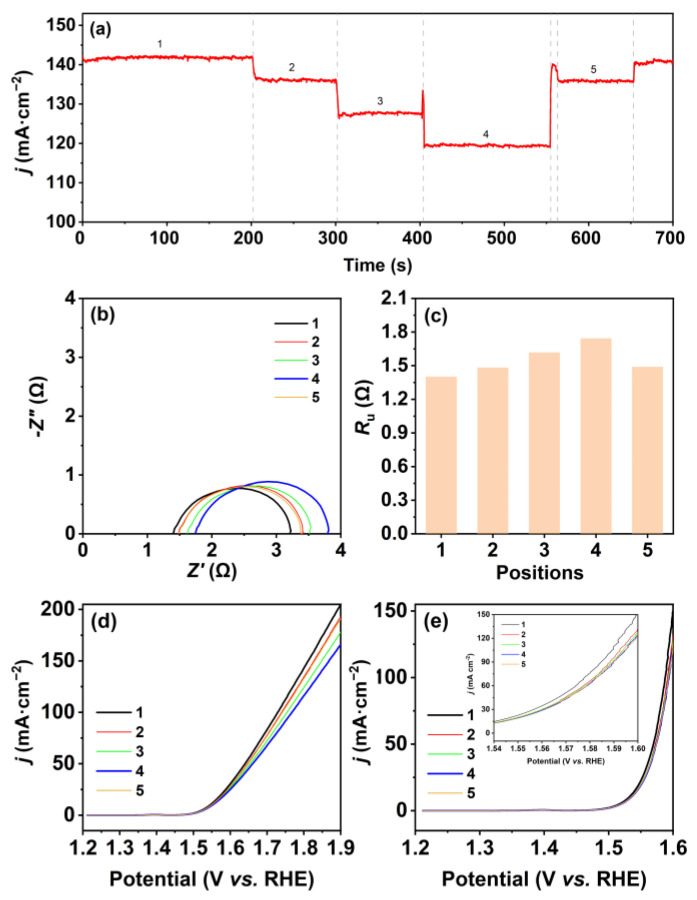
(**a**) Chronoamperometric curve for the WE at different positions in the small electrolytic container at 0.9 V (vs. Hg/HgO electrode); (**b**) Nyquist plots of the WE and (**c**) the responding *R*_u_ at different positions in the small electrolytic container; OER polarization curves of the WE (**d**) without *iR*_u_ compensation and (**e**) with *iR*_u_ compensation for the WE at different positions in the small circular container after chronoamperometry. Note: the number 1–5 refer to the numbers of different positions.

The changes in current density were recorded through the chronoamperometric curve while moving. As shown in Figure 2a, the current density is the highest when the three-electrode system is put at position 1, followed by a 3.5% decrease at position 2, where the WE is located in the center of the container. Another decline in current density with 6.6% is found at position 3, where the CE is placed parallel to and adjacent to the container wall. The current density drops to the lowest value when the WE and CE are located vertically close to the container wall (position 4) and then increased to a similar level to that at position 5, where the RE is near the container wall. The current density can be restored to its initial level when the electrode is relocated back to position 1. This trend was consistent with the results of polarization curves before *iR*_u_ compensation. As shown in Figure 2d, the polarization curve of the WE at position 1 is located at the top, while the polarization curve of the WE at position 5 is situated at the bottom. Additionally, the polarization curve of the WE at position 3 is positioned below the curves of the WE at position 2 and 4. *R*_u_ is responsible for correcting the uncompensated potential drop due to the ionic current flowing through the electrolyte as between the WE and the RE [28]. We suspect the Ru value in different locations of the electrolytic container may vary due to the concentration boundary layer of electrolyte. So, we obtained their corresponding *R*_u_ values from Nyquist plots (Figure 2b), which are listed in Figure 2c. The *R*_u_ reaches its peak at position 1 with minimum current density and, conversely, the *R*_u_ is at its lowest at position 4, experiencing the highest current density. After *iR*_u_ corrected, the differences in the potential between different positions have significantly decreased, as exhibited in Figure 2e. This indicates that the differences in potentials at different positions are mainly caused by *R*_u_. In addition, a slight difference is still observed in overpotential between the electrodes at position 1 and the electrodes at other positions, which can be ascribed to their different gas diffusion resistances.

**Figure 3:**
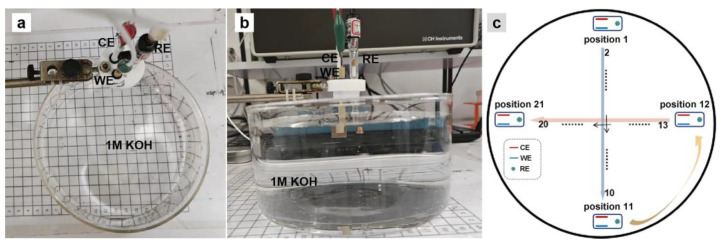
(**a**) Top-view photo and (**b**) side-view photo of the three-electrode system in the big circular container; (**c**) the position trajectory of the three-electrode system in the big circular container (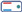: the three-electrode system).

**Figure 4:**
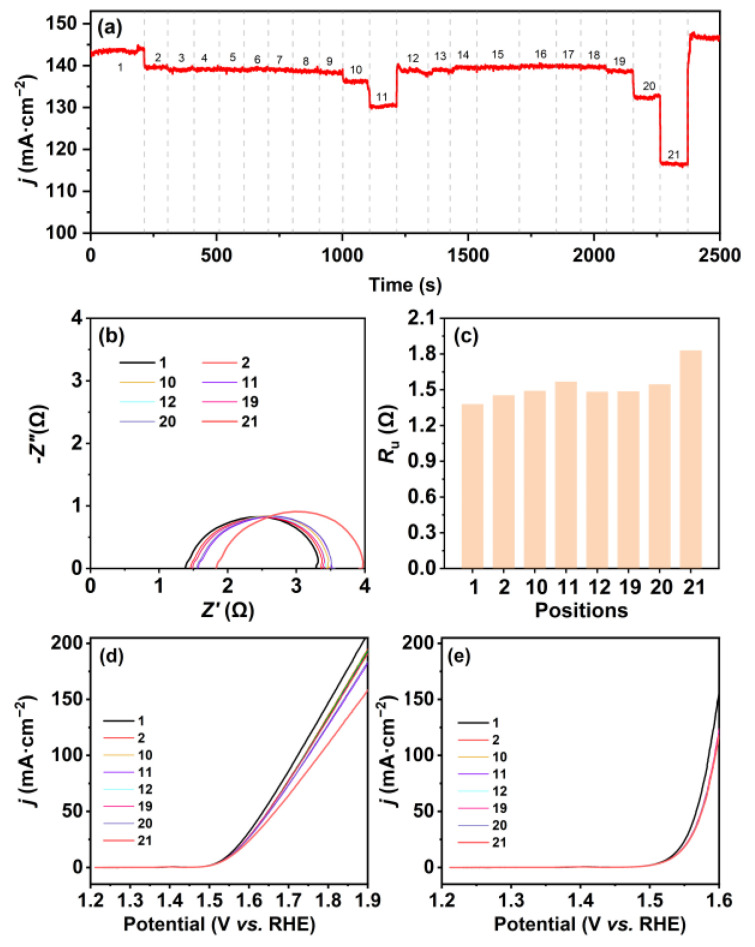
(**a**) Chronoamperometric curve for the WE at different positions in the big electrolytic container at 0.9 V (vs. Hg/HgO electrode); (**b**) Nyquist plots of the WE and (**c**) the responding *R*_u_ at different positions in the big circular container; OER polarization curves of the WE (**d**) without *iR*_u_ compensation and (**e**) with *iR*_u_ compensation for the WE at different positions in the big circular container after chronoamperometry. Note: the numbers 1–21 refer to the numbers of different positions.

Because of the constrained space within the small electrolytic container and the limited availability of adjustable positions for the three-electrode system, we then employed a bigger container with a diameter of ~12.5 cm and a height of ~7.5 cm to investigate the changes in the current density at different positions (Figure 3a,b). The position trajectory of the electrode system in the bigger electrolytic container is shown in Figure 3c. As expected, the chronoamperometric curve shows multiple steps with the current density changing from 145 to 116.6 mA cm^−2^ when moving in the electrolytic container (Figure 4a). The current density starts at its peak when the electrode system is at position 1, where the WE and CE lie on the central axis and the CE is near the container wall. A drop of 2.7% in the current density is found when the electrodes move to position 2, followed by infinitesimal fluctuation around 138 mA cm^−2^ from position 3 to position 9. Then, the current density decreases by 1.4% at position 10 and further decreases by 4.4% at position 11, which is the opposite of position 1, with the WE near the container wall. The current density recovered to 138 mA cm^−2^ when the electrodes move to position 12, where the RE is near the container wall whereas the WE and CE are vertical to the wall. Prior to the electrodes reaching position 19, the current density undergoes marginal fluctuations. The current density decreases by 4.3% at position 20 and, subsequently, it drops by 12% to reach its lowest value at position 21, where the WE and CE are vertically positioned close to the container wall. The results align with the variation trend of polarization curves prior to *iR*_u_ compensation, as well as being in line with the results of small electrolytic container experiments (Figure 2a and Figure 4d).

As expected, the variation in *R*_u_ is likewise in agreement with the results from the small electrolytic container experiments, i.e., the resistance is lowest at the position that shows the highest current density and vice versa. The values of *R*_u_ and the polarization curves corrected by *iR*_u_ are shown in Figure 4c,e. It can be seen that the fluctuations in current density that occur at different positions during the chronoamperometry test are mainly attributed to alterations in *R*_u_ magnitude; therefore, the polarization curves after *iR*_u_ compensation are nearly coincident for the WE at most positions, except for position 1. The *R*_u_ can be calculated by Equation (1) for a simple planar electrode:

(1) $$R_{u}=\frac{x}{\kappa A}$$

where *x* is the distance from the tip of RE to WE, *A* is the area of the WE, and *κ* is the solution conductivity [28,29]. When the distance from the tip of RE to WE and the area of the WE are given fixed values, the *R*_u_ become affected by the solution conductivity only. In this work, we treated the three-electrode system as a whole and adjusted the position of the electrode system in the container via moving the container itself, so the distance of the RE tip to WE and the area of the WE are constants; therefore, the *R*_u_ variation in different positions in the container is mainly caused by the conductivity of the electrolyte solution in the container, which is related to the concentration of ions in the electrolyte solution [30].

According to the theory of the concentration boundary layer, when a fluid flows past a solid wall, a concentration gradient exists within the fluid in the vertical direction of the wall if there is a dissimilarity between the concentration of a component in the fluid and that of the solid wall [31,32], and the concentration gradient diminishes progressively from the wall towards the mainstream liquid [33]. This results in the variations in *R*_u_ at different positions in the container and higher *R*_u_ in positions closer to the container wall. From the results of the *iR*_u_ corrected polarization curves, a majority of curves are overlapped by the compensation (Figure 2e and Figure 4e). However, there are certain curves located above or below the overlapped curves (e.g., the curves of position 1 in the small or big circular container) as a result of the gas diffusion resistance, which can hinder the attainment of accurate and reliable data and interfere with the fair comparison of materials’ overpotential for OER.

**Figure 5:**
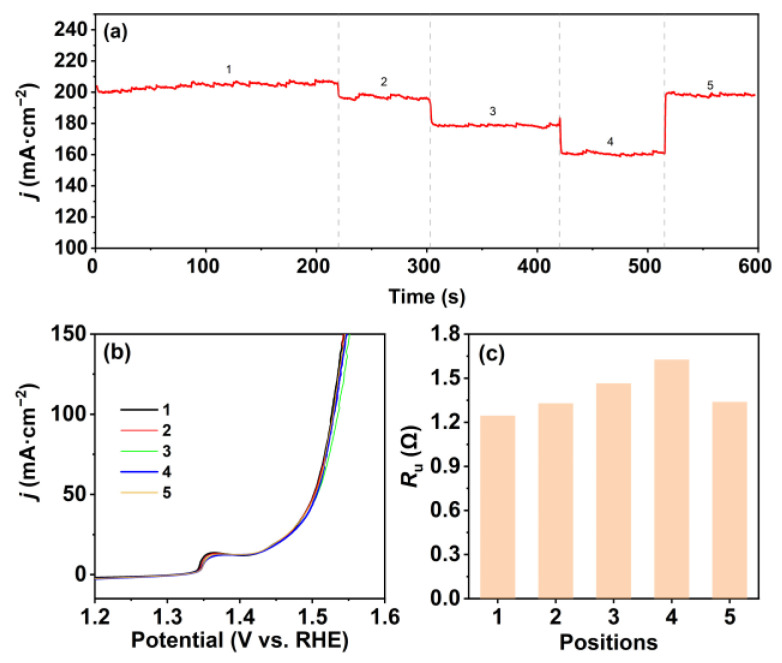
(**a**) Chronoamperometric curve for the NiFeLDH at different positions in the small electrolytic container at 0.9 V (vs. Hg/HgO electrode); (**b**) OER polarization curves of the NiFeLDH with *iR*_u_ compensation and (**c**) the responding *R*_u_ at different positions in the small circular container after chronoamperometry. Note: the numbers 1–5 refer to the numbers of different positions.

In order to verify the generalization of this phenomenon, we attempted to use NiFe-layered double hydroxide (LDH) grown on Ni foam as the WE, Hg/HgO electrode as the RE, a Pt mesh as the CE, and 1 M NaOH solution as the electrolyte to investigate the fluctuations in current density during the electrode’s movement, and the position trajectory of the three-electrode system in the small circular container was the same as the trajectory in Figure 1d. As show in Figure 5, the electrochemical test results further prove that the variations in *R*_u_ at different positions are the primary factor behind the fluctuations in current density during movement. Hence, for fair comparisons and reliable results, it is advisable to place the electrodes in the central region of the electrolytic container and maintain the electrode positions unchanged throughout electrochemical experiments. It is important to emphasize the need to evaluate the *R*_u_ whenever there are changes in the container setup during testing.

## 3. Materials and Methods

### 3.1. Materials

Potassium hydroxide (KOH), sodium hydroxide (NaOH), nickel nitrate hydrate (Ni(NO_3_)_2_·6H_2_O), ferric nitrate hydrate (Fe(NO_3_)_3_·9H_2_O), ammonium fluoride (NH_4_F), urea (CH_4_N_2_O), acetone, and ethanol were purchased from Sinopharm Chemical Reagent Co., Ltd. (Shanghai, China), which were directly used without further purification. Ni sheets and Ni foam were obtained from Hefei Wenghe Metal Materials Co., Ltd. (Hefei, China). Ni sheets were cleaned with acetone, ethanol, and deionized water for 30 min each before use as electrodes, and Ni foams were pretreated with sonication for 10 min in acetone, hydrochloric acid solution (1 M), deionized water, and ethanol before use.

NiFe-LDH was grown on Ni foam by hydrothermal method according to the previous work [34]. Typically, Ni(NO_3_)_2_·6H_2_O (0.291 g), Fe(NO_3_)_3_·9H_2_O (0.404 g), NH_4_F (0.224 g), and urea (0.6 g) were dissolved in 30 mL deionized water under stirring. The clear solution and Ni foams were transferred into a 50 mL Teflon-lined stainless-steel autoclave for solvothermal treatment at 120 °C for 12 h, and then the products were washed with water and dried at 70 °C in a vacuum oven.

### 3.2. Electrochemical Characterizations

Electrochemical measurements were performed in an electrolytic container with a three-electrode system at room temperature (~298 K) by using an electrochemical workstation (CHI 760E). The working electrode was a Ni sheet or NiFeLDH with dimensions of 1 cm × 1 cm, the reference electrode was a Hg/HgO electrode, and the counter electrode was a Pt mesh. OER performance of the Ni sheet was tested in a 1.0 M KOH solution. Prior to conducting the electrochemical measurements, 100 cyclic voltametric (CV) scans were performed to activate the working electrode at a scan rate of 50 mV s^−1^ within the voltage range of 0.3 to 1.0 V (vs. RHE). After electrochemical preactivation, the current change at different positions of the electrode was observed through chronoamperometry performed at 0.9 V (vs. Hg/HgO electrode). The values of *R*_u_ at different positions were tested by electrochemical impedance spectroscopy (EIS) measurements, which were carried out at 0.65 V (vs. Hg/HgO electrode) from 100 kHz to 0.1 Hz. Polarization curves were obtained using LSV at a scan rate of 2 mV s^−1^. The measured potentials were converted to the RHE scale, according to Equation (2), and the potential drops generated from *R*_u_ were manually corrected by following Ohm’s law, according to Equation (3):

E_RHE_ = E_Hg/HgO_ + 0.0592 pH + 0.098 V, (2)

E_RHE (corrected)_ = E_RHE (uncorrected)_ − *iR*_u_, (3)

## 4. Conclusions

We have employed a movable electrolyte container setup to gain insights into the effects of electrode positions in the electrolytic container for the accurate evaluation of an OER electrocatalyst. The results indicate that the current density fluctuations when adjusting the electrodes’ position in the container are primarily caused by their different *iR*_u_ drop and the gas diffusion resistance. The *R*_u_ values at different positions, along with the recorded LSV curves before and after performing *iR*_u_ compensation, unveil a clear correlation between *R*_u_ and the position of the electrodes in the container. We also observed that the gas diffusion is significantly higher when the electrode is placed parallel to the container compared to the positions vertical to the container wall. For accurate data analysis and fair comparisons, the electrode positions are highly recommended to be fixed in the central region in the electrolytic container and, if any movement occurs, it will be necessary to remeasure the resistance. Moreover, it is imperative to establish a standard electrolyzer setup for electrochemical assessments in the electrochemical community.

## Data Availability

Data are contained within the article.

## References

[1] Hassan Q., Sameen A.Z., Salman H.M., Jaszczur M., Al-Jiboory A.K. (2023). Hydrogen energy future: Advancements in storage technologies and implications for sustainability. J. Energy Storage.

[2] Megía P.J., Vizcaíno A.J., Calles J.A., Carrero A. (2021). Hydrogen Production Technologies: From Fossil Fuels toward Renewable Sources. A Mini Review. Energy Fuels.

[3] Wang Y., Vogel A., Sachs M., Sprick R.S., Wilbraham L., Moniz S.J.A., Godin R., Zwijnenburg M.A., Durrant J.R., Cooper A.I. (2019). Current understanding and challenges of solar-driven hydrogen generation using polymeric photocatalysts. Nat. Energy.

[4] Zhu J., Hu L., Zhao P., Lee L.Y.S., Wong K.-Y. (2020). Recent Advances in Electrocatalytic Hydrogen Evolution Using Nanoparticles. Chem. Rev..

[5] Terlouw T., Bauer C., McKenna R., Mazzotti M. (2022). Large-scale hydrogen production via water electrolysis: A techno-economic and environmental assessment. Energy Environ. Sci..

[6] Kibsgaard J., Chorkendorff I. (2019). Considerations for the scaling-up of water splitting catalysts. Nat. Energy.

[7] Song J., Wei C., Huang Z.-F., Liu C., Zeng L., Wang X., Xu Z.J. (2020). A review on fundamentals for designing oxygen evolution electrocatalysts. Chem. Soc. Rev..

[8] Li H., Pan Y., Wu L., He R., Qin Z., Luo S., Yang L., Zeng J. (2023). IrO_2_ deposited on RuO_2_ as core-shell structured RuO_2_@IrO_2_ for oxygen evolution reaction in electrochemical water electrolyzer. Mol. Catal..

[9] Wu Z.-Y., Chen F.-Y., Li B., Yu S.-W., Finfrock Y.Z., Meira D.M., Yan Q.-Q., Zhu P., Chen M.-X., Song T.-W. (2023). Non-iridium-based electrocatalyst for durable acidic oxygen evolution reaction in proton exchange membrane water electrolysis. Nat. Mater..

[10] Qin Y., Yu T., Deng S., Zhou X.-Y., Lin D., Zhang Q., Jin Z., Zhang D., He Y.-B., Qiu H.-J. (2022). RuO_2_ electronic structure and lattice strain dual engineering for enhanced acidic oxygen evolution reaction performance. Nat. Commun..

[11] Mamaca N., Mayousse E., Arrii-Clacens S., Napporn T.W., Servat K., Guillet N., Kokoh K.B. (2012). Electrochemical activity of ruthenium and iridium based catalysts for oxygen evolution reaction. Appl. Catal. B Environ..

[12] Yang L., Liu Z., Zhu S., Feng L., Xing W. (2021). Ni-based layered double hydroxide catalysts for oxygen evolution reaction. Mater. Today Phys..

[13] Khan N.A., Rashid N., Junaid M., Zafar M.N., Faheem M., Ahmad I. (2019). NiO/NiS Heterostructures: An Efficient and Stable Electrocatalyst for Oxygen Evolution Reaction. ACS Appl. Energy Mater..

[14] He D., Song X., Li W., Tang C., Liu J., Ke Z., Jiang C., Xiao X. (2020). Active Electron Density Modulation of Co_3_O_4_-Based Catalysts Enhances their Oxygen Evolution Performance. Angew. Chem. Int. Ed..

[15] Li S., Hao X., Abudula A., Guan G. (2019). Nanostructured Co-based bifunctional electrocatalysts for energy conversion and storage: Current status and perspectives. J. Mater. Chem. A.

[16] Xu X., Su C., Shao Z. (2021). Fundamental Understanding and Application of Ba_0.5_Sr_0.5_Co_0.8_Fe_0.2_O_3−δ_ Perovskite in Energy Storage and Conversion: Past, Present, and Future. Energy Fuels.

[17] Xu X., Wang W., Zhou W., Shao Z. (2018). Recent Advances in Novel Nanostructuring Methods of Perovskite Electrocatalysts for Energy-Related Applications. Small Methods.

[18] Nong H.N., Falling L.J., Bergmann A., Klingenhof M., Tran H.P., Spöri C., Mom R., Timoshenko J., Zichittella G., Knop-Gericke A. (2020). Key role of chemistry versus bias in electrocatalytic oxygen evolution. Nature.

[19] Kang W., Wei R., Yin H., Li D., Chen Z., Huang Q., Zhang P., Jing H., Wang X., Li C. (2023). Unraveling Sequential Oxidation Kinetics and Determining Roles of Multi-Cobalt Active Sites on Co_3_O_4_ Catalyst for Water Oxidation. J. Am. Chem. Soc..

[20] Huang Z.-F., Song J., Du Y., Xi S., Dou S., Nsanzimana J.M.V., Wang C., Xu Z.J., Wang X. (2019). Chemical and structural origin of lattice oxygen oxidation in Co–Zn oxyhydroxide oxygen evolution electrocatalysts. Nat. Energy.

[21] Righi G., Plescher J., Schmidt F.-P., Campen R.K., Fabris S., Knop-Gericke A., Schlögl R., Jones T.E., Teschner D., Piccinin S. (2022). On the origin of multihole oxygen evolution in haematite photoanodes. Nat. Catal..

[22] Haase F.T., Bergmann A., Jones T.E., Timoshenko J., Herzog A., Jeon H.S., Rettenmaier C., Cuenya B.R. (2022). Size effects and active state formation of cobalt oxide nanoparticles during the oxygen evolution reaction. Nat. Energy.

[23] Wang X., Xi S., Huang P., Du Y., Zhong H., Wang Q., Borgna A., Zhang Y.-W., Wang Z., Wang H. (2022). Pivotal role of reversible NiO_6_ geometric conversion in oxygen evolution. Nature.

[24] Jia H., Yao N., Yu C., Cong H., Luo W. (2023). Unveiling the Electrolyte Cations Dependent Kinetics on CoOOH-Catalyzed Oxygen Evolution Reaction. Angew. Chem. Int. Ed..

[25] Zhao K., Tao Y., Fu L., Li C., Xu B. (2023). Bifunctional Near-Neutral Electrolyte Enhances Oxygen Evolution Reaction. Angew. Chem. Int. Ed..

[26] Liang Q., Brocks G., Bieberle-Hütter A. (2021). Oxygen evolution reaction (OER) mechanism under alkaline and acidic conditions. J. Phys. Energy.

[27] Tang J., Xu X., Tang T., Zhong Y., Shao Z. (2022). Perovskite-Based Electrocatalysts for Cost-Effective Ultrahigh-Current-Density Water Splitting in Anion Exchange Membrane Electrolyzer Cell. Small Methods.

[28] Stevens M.B., Enman L.J., Batchellor A.S., Cosby M.R., Vise A.E., Trang C.D.M., Boettcher S.W. (2017). Measurement Techniques for the Study of Thin Film Heterogeneous Water Oxidation Electrocatalysts. Chem. Mater..

[29] Bard A.J., Faulkner L.R. (2001). Electrochemical Methods: Fundamentals and Applications.

[30] Zhang W., Chen X., Wang Y., Wu L., Hu Y. (2020). Experimental and Modeling of Conductivity for Electrolyte Solution Systems. ACS Omega.

[31] Pérez-Herranz V., Guiñón J.L., García-Antón J. (2000). A new technique for the visualization of the concentration boundary layer in an electrodialysis cell. J. Appl. Electrochem..

[32] Zhou Y. (2011). Materials Engineering Fundamentals.

[33] Zhu M., Yu Q. (2014). Thermal Engineering Fundamentals.

[34] Wu B., Gong S., Lin Y., Li T., Chen A., Zhao M., Zhang Q., Chen L. (2022). A Unique NiOOH@FeOOH Heteroarchitecture for Enhanced Oxygen Evolution in Saline Water. Adv. Mater..

